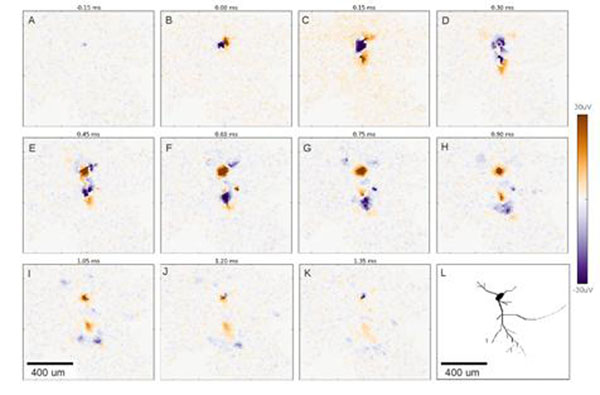# High-resolution mapping of single neurons provides insight into neuron structure and LFP generation

**DOI:** 10.1186/1471-2202-12-S1-P75

**Published:** 2011-07-18

**Authors:** Patrick Dini, Maxime Ambard, Ulrich Egert, Urs Frey, Andreas Hierlemann

**Affiliations:** 1 Bernstein Center Freiburg, Albert-Ludwigs-University Freiburg, Freiburg 79100, Germany; 2 Institute of Biology III, Albert-Ludwigs-University Freiburg, Freiburg 79100, Germany; 3 Biomicrotechnology, Department of Microsystems Engineering, Albert-Ludwigs-University Freiburg, Freiburg 79100, Germany; 4 Bio Engineering Laboratory, Department of Biosystems Science and Engineering, ETH Zurich, Basel 4058, Switzerland

## 

Recent modeling [1] has suggested that the spatial structure of single neurons, especially the orientation and the shape of their dendritic trees, is of great importance in the understanding of the properties of the LFP generated (for example, a low-pass filtering effect has been shown in remote neurites [2]). In order to test these predictions, high-density microelectrode arrays (MEAs) featuring 11'011 electrodes are a valuable tool [3]. They provide detailed information about the external electrical field potentials of cultured neurons, from which the relevant information about single neurons properties must be extracted. We developed an on-line software allowing us to track neurites of single neurons (Figure 1A-K, footprint of a neuron), which provides information about their spatial structure and their activity dynamics leading to predictions on their morphology (Figure 1L). These allow us to elucidate additional properties of LFP generation, such as, multi-polar potentials related to the morphology of the studied cell. Moreover, reconstruction of the morphology of different cells was performed based on footprints and compared with imaging from GFP-stained neural cultures.

**Figure 1:**